# Status of post-crash response systems and policy indicators in the WHO African region: a cross-sectional descriptive study

**DOI:** 10.1136/bmjph-2025-004541

**Published:** 2026-10-09

**Authors:** Binta Sako, Eunice Nahyuha Chomi, Chiara Retis, Olushayo Oluseun Olu, Joseph Okeibunor, Adelheid Onyango

**Affiliations:** 1 World Health Organization Regional Office for Africa, Brazzaville, Brazzaville, Congo; 2 Independent Researcher, Dakar, Senegal; 3 Emergency Preparedness and Response, World Health Organization Regional Office for Africa, Brazzaville, Brazzaville, Congo; 4 Department of Sociology/Anthropology, University of Nigeria, Nsukka, Nigeria; 5 Health Systems and Services Cluster, World Health Organization Regional Office for Africa, Brazzaville, Brazzaville, Congo

**Keywords:** Accidents, Traffic, Emergencies, Public Health, Health Services Accessibility

## Abstract

**Introduction:**

The WHO African region has the highest estimated road traffic fatality rate globally. Effective post-crash response and care are essential to mitigate the severity and fatality of injuries from road traffic crashes. Post-crash response can be conceptualised as a continuum of emergency care functions spanning the scene of injury, prehospital and facility-based care. This study describes the reported fatality-to-crash ratio and the status of post-crash response system and policy indicators in the WHO African region using the most recent data available. It identifies the gaps to inform the strengthening of post-crash response systems in the region.

**Methods:**

We conducted a cross-sectional descriptive analysis using data from the Global Status Report on Road Safety 2018 and 2023 databases. Indicators were selected to reflect key components of the post-crash continuum, including prehospital services, facility-based emergency care and system-level enabling factors. We analysed the reported fatality-to-crash ratio and the availability of selected policy and system indicators related to post-crash response and care. Comparisons between 2018 and 2023 were conducted for a subset of comparable indicators. The analysis is descriptive and does not assess policy implementation or effectiveness.

**Findings:**

Across the 40 countries reporting complete data, 238 851 crashes resulted in 340 170 injuries and 50 993 deaths, corresponding to a reported regional fatality-to-crash ratio of 21% with substantial variability observed across countries, including values exceeding 100%, which may reflect differences in reporting systems, under-reporting of non-fatal crashes and the occurrence of crashes involving multiple fatalities. Indicators showed limited reported availability of prehospital services, uneven access to emergency care facilities and shortages of trained personnel. Free psychological support and rehabilitation services were rarely available. Between 2018 and 2023, most comparable indicators showed declines in reported availability. However, interpretation of these findings is limited by substantial missing data for several indicators and potential inconsistencies in reporting across countries.

**Conclusion:**

The findings suggest important gaps in post-crash response systems across the WHO African Region, particularly in prehospital care, emergency service capacity and rehabilitation. Strengthening emergency care systems and improving the quality and integration of injury data systems are important priorities for strengthening post-crash response in the region. Further research is needed to assess policy implementation and barriers to effective post-crash care.

**WHAT IS ALREADY KNOWN ON THIS TOPIC**

- Road traffic injuries are the leading cause of morbidity and mortality among people aged 5 to 29 and ranks 11^th^ across all ages in the WHO African region. Poor post-crash response and care are considered important contributors to preventable mortality and disability following road traffic crashes.
- While substantial research has focused on road crash prevention strategies across the African region, the critical domain of post-crash response and care has been examined as part of broader emergency medical services research, with most analyses conducted at the country level.
- Existing literature generally describes post-crash response systems in many African settings as fragmented, resource-constrained and characterised by important urban–rural disparities in access to and quality of care.

**WHAT THIS STUDY ADDS**

- This assessment provides a regional overview of the key post-crash response and care system indicators across 45 countries in the WHO African region that participated in the Global Status Report on Road Safety published in 2023, including selected legislative and policy measures related to post-crash response.
- The assessment compares selected indicators between the 2018 and 2023 reporting periods and suggests declines in the reported availability of several key post-crash response and care indicators, although interpretation is limited by data availability and comparability constraints.

**HOW THIS STUDY MIGHT AFFECT RESEARCH, PRACTICE OR POLICY**

- The findings suggest important gaps in post-crash response and care systems in many African countries and highlight the need to strengthen emergency care systems, rehabilitation services and injury surveillance systems.
- The findings also highlight the need for further research on barriers to implementation of post-crash response policies and services, including context-specific factors influencing access to and quality of care.

## Introduction

 Road traffic crashes are a significant public health concern worldwide, killing 1.19 million people each year, and are the leading killer of children and youth 5–29 years.^1^ The WHO African Region, comprised of 47 member states, is experiencing a disproportionate burden. According to WHO estimations, the region accounted for 19% of global road traffic deaths in 2021, despite having 15% of the world population and only about 3% of the world’s vehicles.^1,2^ For over a decade, the region has recorded the highest fatality rate for road traffic deaths in the world. It is also the only region in over 10 years that has witnessed an increase by 17% in the number of road deaths.^1,2^ The latest WHO estimates show that road traffic fatalities rates are 19.4 per 100 000 compared to 15 per 100 000 globally.^1,2^ Vulnerable road users such as pedestrians, cyclists and motorcyclists account for 50% of all deaths. Road deaths predominantly affect men in their economically active years, and pedestrians alone account for one-third of fatalities, the highest in the world.^2^ The high rates of fatalities and injuries in the region are associated with several factors, including rapid and unplanned urbanisation, poor road infrastructure, poor vehicle regulations, insufficient provision of traffic regulations and traffic law enforcement, and limitations in post-crash response systems. Road crashes not only lead to loss of life but also cause long-term disabilities, economic losses and psychological trauma for victims and their families. Globally, countries lose between 1% and 3% of their gross domestic product (GDP) to road crashes every year.^1^ The economic consequences are estimated to be worse for Sub-Saharan Africa where GDP loss is estimated to be between 0.8% and 9%. For some countries, this loss is more than the total aid they receive annually.^3^ A recent study conducted in five low- and middle-income countries, two of which are from the WHO African region, further demonstrates the long-term impacts of road crashes and insufficiency of care from medical emergency services to rehabilitation, leading to increased disabilities and requirements for long-term care. The study highlights gender inequities, showing that although men are more affected by crashes (81% in the study), women are more susceptible to negative outcomes, experiencing significantly higher barriers to accessing healthcare and recovery services than men after a crash.^4^

Under WHO leadership, the international community produced a Global Plan for the Decade of Action for Road Safety 2021–2030^5^ to address the epidemic of road traffic injuries and deaths. The plan provides a framework for countries to improve road safety and achieve the sustainable development goal (SDG) target 3.6 of halving road traffic deaths and injuries by 2030. It is based on five domains: road safety management, safer roads and mobility, safer vehicles, making road users safer and improved post-crash response and care.^5^

Effective post-crash response is crucial for minimising the consequences of road traffic crashes.^6^ The decline in road traffic deaths and the severity of injuries witnessed in many high-income countries can be attributed to the improvements in trauma care.^7,8^ A recent study reported that improvements in trauma care could save more than a million lives, cutting road injury deaths by up to 30% in developing countries.^9^

Post-crash response can be conceptualised as a continuum of emergency care functions spanning the scene of injury, prehospital transport, facility-based emergency care and early inpatient management. The WHO Emergency Care System Framework^10^ provides a structured approach to analysing these components and the enabling system factors that support them, including governance, workforce, infrastructure, financing and information systems (figure 1). In this study, we draw on this framework to examine the availability of key elements of post-crash response systems in the WHO African Region.

**Figure 1:**
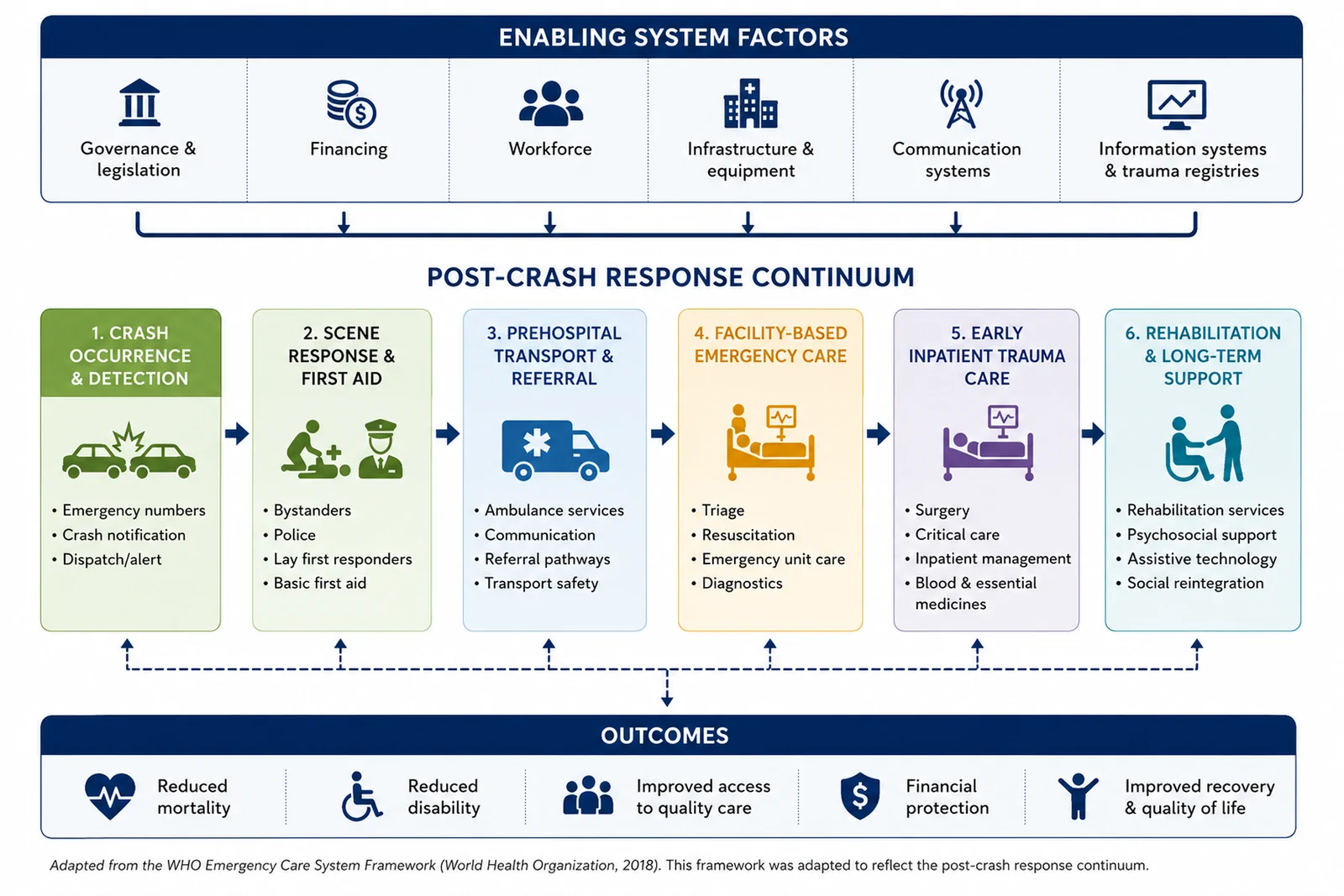
Conceptual framework for post-crash response in the WHO African Region, adapted from the WHO Emergency Care System Framework.

Several studies have examined the epidemiology of road traffic crashes in the WHO African Region, focusing on factors such as injury patterns, risk factors and preventive measures.^11–13^ However, despite growing literature on emergency medical services in Africa, post-crash interventions are often included as part of the broader emergency system analyses. Relatively limited research has specifically examined post-crash response systems, policy indicators and emergency care capacity at a regional level.^14^ Existing literature often highlights deficiencies in emergency medical services, including delays in response times, lack of trained personnel and inadequate medical facilities.^6^ A systematic review of prehospital emergency care in low- and middle-income countries found that prehospital care is fragmented and uncoordinated, lacking trained medical personnel and first responders, inadequate basic materials and substandard infrastructure.^15^

This study aims to describe the status of post-crash response systems and related policy indicators in the WHO African Region. It also highlights reported gaps and areas requiring strengthening. The findings aim to inform policymakers, healthcare providers and stakeholders on priorities to improve post-crash response systems in the region.

## Materials and methods

### Study design and area

We conducted a cross-sectional descriptive study of the post-crash response and care to road traffic crashes using secondary data from the WHO African Region. Data were collected from the Global Status Report on Road Safety database of 2023 in which 45 countries participated and the Global Status Report on Road Safety database of 2018 in which 44 countries participated. 42 countries participated in both reports. The list of countries that participated in each report is presented in online supplemental materials 1.

### The global status report on road safety database

The 2023 Global Status Report on Road Safety database contains country-reported data from 172 countries and territories. Data were gathered through a self-administered standardised online questionnaire. The questionnaire was based on the one produced for the first Global Status Report in 2009 and was expanded and revised to better reflect the Decade of Action for Road Safety 2021–2030 framework.^5^ Therefore, the survey covers road safety governance, transport modalities, road user behaviour, road infrastructure, vehicle safety and post-crash response and care policies and key indicators. The questionnaire was translated and available in all six UN languages.

The data collection process began from September 2022 to August 2023. Every country was invited to nominate a national data focal person (NDFP) who, in turn, sought up to 10 multisectoral collaborators (NDCs) contributions to complete the online survey. The contributors were expected to be experts in the field. Countries were requested to provide data for 2021 or the closest available. An iterative review and validation process between the NDFP, the WHO regional advisors and WHO headquarters followed until the final sign-off of the questionnaire by countries. Data were assessed for internal consistency, accuracy and completeness during the validation process. Internal consistency checks included verification of adherence to skip patterns and comparison with data from previous survey rounds. The accuracy of responses, particularly regarding the availability of post-crash response elements, was verified against official country documents, including national policies, strategic plans, reports, statistical records and relevant legislation. Follow-up was conducted with countries that submitted incomplete responses, and the response options ‘no response’ or ‘don’t know’ were used only as a last resort after confirmation with the respective countries. While this iterative validation process strengthens the consistency of the data, it should be noted that the information remains largely based on country-reported inputs and may be subject to reporting bias. The data reflects the knowledge and perspectives of national focal points and contributors and may not fully capture variations in implementation or service availability within countries.

We have also used the 2018 Global Status Report database to assess change. This database was built using the same methodology as previously described. For this database, the countries were requested to provide data for 2017 or the closest available. For this assessment, we will refer to the year of the report rather than the year of the data. In addition to the data collected through the standardised questionnaires, both databases included data on populations from the UN Population Division and World Bank income level classification, data on UN road safety conventions and data on road density and safety score ratings from the International Road Federation and International Road Assessment Programme.

### Data collection and analysis

The Global Status Report questionnaires include indicators relevant to post-crash which were selected based on the WHO Emergency Care System Framework, allowing a structured assessment of post-crash response across the continuum of care and associated system components.

From the 2023 database, we identified and extracted data relating to crashes, injuries and deaths as reported by countries and policy measures relevant to post-crash response and care. The policy measures included having legislation on universal access to emergency care, a national emergency number, formal certification for prehospital care, access to adequate prehospital care in urban and rural areas, availability of medical and nurse specialists, conducting formal assessments of emergency care, availability of trauma registries, psychological support free of charge, rehabilitation services free of charge and national legislation mandating third-party liability insurance for powered vehicles. We quantitatively analysed the data and presented frequency distributions by country, income level and subregion when relevant. Some indicators included combined ‘Do not know/no response’ categories in the original questionnaire, limiting the ability to distinguish between respondent uncertainty and incomplete reporting. For indicators assessing the distribution and accessibility of emergency care services, countries selected the questionnaire response option that best described their national situation. Terms such as ‘adequate coverage’ reflect predefined response categories included in the WHO Global Status Report on Road Safety questionnaire and represent country-reported assessments. These predefined categories were retained in the analysis to ensure consistency with the original survey instrument. We calculated a reported fatality-to-crash ratio, defined as the number of reported deaths divided by the number of reported crashes and expressed as a percentage. This indicator reflects the relationship between reported fatalities and crash events rather than deaths among injured individuals. As a single crash may involve multiple fatalities, values exceeding 100% are theoretically possible. However, substantial under-reporting of non-fatal crashes and differences in reporting systems across countries may further influence the observed ratios.

To assess progress, we extracted data from the 2018 database that were relevant to post-crash response and care. Due to differences in questionnaire content between the 2018 and 2023 Global Status Report databases, comparisons were limited to indicators that were available and similarly defined in both reporting periods. We identified six indicators considered sufficiently comparable across the two datasets: single national emergency number, trauma registries (national/subnational/facility based), assessment of the emergency system, doctors with medical specialty, doctors with trauma specialty and nurses with training in trauma/emergency care. However, differences in reporting practices, respondent interpretation and participating countries may still influence comparability across reporting periods.

A full and detailed description of the data collection methodology to build the database is in Annex 1 of the Global Status Report on Road Safety 2023^1^ and in the Explanatory Note 1 of the Global Status Report on Road Safety 2018.^16^ Data used in this study were derived from the WHO Global Status Report on Road Safety 2023 and 2018 databases, which were developed by WHO using standardised country-level data submitted by designated national focal points and validated through the Global Status Report on Road Safety data collection process. WHO-estimated fatalities also presented in these databases are derived directly from vital registration data where these are at least 80% complete. Elsewhere, WHO uses a negative binomial regression model with population as the exposure and covariates including GDP, vehicle ownership, road density, speed limits, alcohol consumption and health system access. Estimates are averaged across the three best-performing models, with CIs.

A subset of indicators collected through the 2023 survey is publicly available through the WHO online Global Status Report on Road Safety (dataset)^17^ and Global Health Observatory portal.^18^ Additional country-level data used in this analysis, including post-crash indicators, are held by the WHO and are not publicly available. The full set of indicators used in this study can be obtained from WHO on reasonable request to sam@who.int, subject to WHO data access procedures.

## Results

### Road crashes, injuries, deaths and reported fatality-to-crash ratio in the WHO African region

Total crashes, injuries and deaths were reported by 40 countries out of the 45 participating in the 2023 Global Status Report. According to the reported data, there were 238 851 crashes, resulting in 340 170 injuries and 50 993 deaths (online supplemental materials 2). There were almost seven times more injuries than deaths. The reported regional fatality-to-crash ratio was 21%, with substantial variability observed across countries, ranging from less than 5% to as high as over 300%. Twenty-five (63%) countries showed a reported fatality-to-crash ratio of less than 25%; however, in five countries, this reported fatality-to-crash ratio was above 50%.

### Existence of laws that govern the availability of emergency care services

Most countries (36 countries, 80%) in the region have an agency coordinating prehospital and emergency care services for the injured.

Ten countries (22%) have universal access to emergency medical care, which refers to specific legislation that ensures access to care regardless of the ability to pay (eg, payment cannot be required as a pre-requisite for receiving care), while double (20 countries, 44%) do not and 15 countries (33%) indicated ‘Do not know’.

When disaggregated by income level, we noted that more countries in the low-middle-income group report having universal access (4 countries out of 10) than the other income groups. However, among low-income countries, substantially more countries reported not having universal access legislation than reported having such legislation (3 vs 13 countries).

### Proportion of the population that has access to a formal prehospital ambulance system

11 of the 45 participating countries (24%) reported having a prehospital ambulance system covering more than 25% of their population, compared with 27% reporting having ambulance services covering less than 25% of the population (table 1).

**Table 1:** Proportion of population covered by a prehospital ambulance system

Proportion of population covered by prehospital ambulance system	Number of countries (n=45)	%
Less than 25% of the population	12	27%
25–50% of the population	1	2%
51–75% of the population	3	7%
76–95% of the population	5	11%
96% or higher of the population	2	4%
Do not know/ data not available	22	49%

Bystanders are often the first responders at the scene of a crash. 17 countries (38%) reported having laws requiring bystanders to help anyone involved in a crash. However, none of the countries provide any protection from civil liability in providing such assistance.

### National emergency access telephone number in countries

All the countries surveyed that provided an answer (37 countries) reported having an emergency number. Of these, 12 countries (32%) have one single number with full national coverage, as recommended. 17 countries (46%) have multiple numbers providing full coverage, and 8 (22%) have limited national coverage.

Six countries report having legislation mandating the availability of Accident Emergency Call Systems (AECS) to trigger an emergency response by a vehicle sensor in all new vehicles.

### Certification for prehospital providers and access to facilities delivering emergency care

Government-ratified certification pathway for prehospital providers may support standardisation and quality of care at the scene of the crash and before access to medical and hospital care. 17 countries (38%) have government-certified pathways for prehospital providers. Only three countries, Mauritius, South Africa and Uganda, provided the number of prehospital providers. South Africa has the highest number and rate with 118.7 per 100 000 population, followed by Mauritius with 7 per 100 000 and Uganda with 0.03 per 100 000 population.

The availability, access and distribution of health facilities providing emergency care suggests geographical disparities between urban and rural areas. 17 countries (38%) report some services in urban areas, although not adequate to population needs, and minimal to no availability of such services in rural settings.

12 other countries report on the uneven distribution of services creating gaps in access. Only two countries, Algeria and Burundi, report adequate emergency care that meets population needs in both urban and rural areas. Table 2 shows the frequency of responses provided by countries on the availability and access to facilities delivering emergency care.

**Table 2:** Distribution of and access to facilities delivering emergency care in WHO Africa region

Availability and access to facilities delivering emergency care	Number of countries n=45	%
There is **some emergency care in urban settings, though not adequate to population needs**; there is no or minimal emergency care availability in rural settings	17	38%
There is adequate emergency care in most urban settings, and moderate emergency care availability in rural settings. **The number and level of emergency care facilities is inadequate or not well distributed for population needs overall**	9	20%
The number and level of emergency care facilities is adequate for population needs overall, but the distribution and coordination between facilities or of access to facilities still leaves gaps in access	3	7%
There is adequate emergency care across the country. **The number, level, distribution, and accessibility of emergency care facilities meets population needs in both urban and rural settings**	2	4%
Do not know/ no response	16	35%

Categories in this table represent predefined response options from the WHO Global Status Report on Road Safety questionnaire. Countries were assigned to the category selected in the survey. Categories are intended to provide broad descriptions of emergency care service availability and distribution and may not capture all aspects of national emergency care capacity

Only seven countries (16%) reported performing national assessments using standardised methods of their prehospital and facility-based emergency care system between 2018 and 2023. These assessments led to the priority actions being conducted in five of these countries. However, 58% of countries report not conducting such assessments, and 27% did not know.

National trauma registries are available in only 24% of countries in the region, while 18% have facility-level registries. Almost 60% of the countries reported ‘Do not know/ no response’ regarding the availability of a national or subnational trauma registry in their country.

Regarding the availability of skilled providers, about half of the countries in the region have medical specialisations (55% in surgery and 47% in emergency care) to provide care to victims of crashes. However, only a third of countries have nurses specialised in emergency/trauma care. (figure 2)

**Figure 2:**
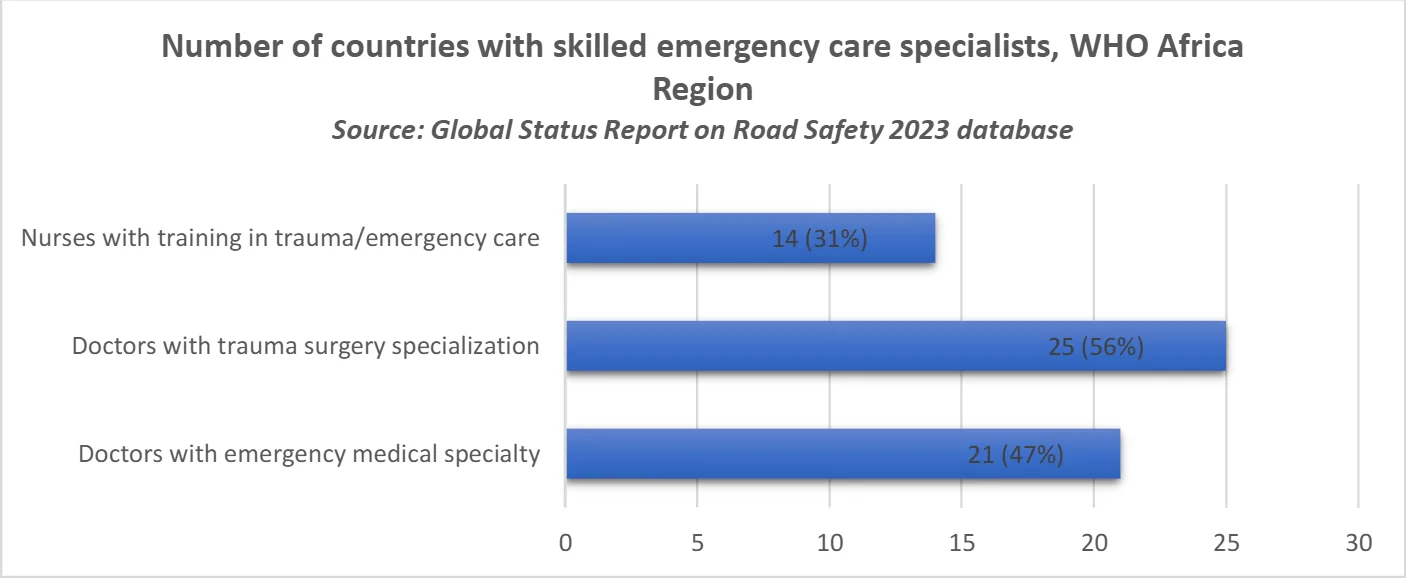
Availability of skilled emergency care specialists.

A substantial proportion of responses across several indicators were categorised as ‘Do not know/no response’. As these categories were combined in the data collection tool, it is not possible to distinguish between lack of information, respondent uncertainty, and incomplete reporting. These findings should therefore be interpreted cautiously, as they may not fully reflect actual system capacity or service availability.

### Long-term impairment, insurance, psychological support, and rehabilitation services

Only three participating countries in the region reported on the estimated long-term impairment caused by road traffic crashes. The reported values range from 3.5% in Namibia to 12.9% in Botswana and 35% in Côte d’Ivoire.

Most countries (36 out of 43 with validated responses, 84%) reported having national legislation on mandatory third-party liability insurance for powered vehicles.

Free access to public/government services for psychological assistance to road victims or their families was unavailable in most countries and only three countries (7%) reported having access to free psychological support. The coverage of such support to survivors was reported by very few countries and was considered low (less than 25% coverage in 2/4 countries that have reported). Similarly, legislation to guarantee free rehabilitative medical care to all injured persons, regardless of their ability to pay is very low with only four countries (9%) reporting availability of services and estimated coverage in three countries reporting coverage of rehabilitation services above 75%.

### Comparison of post-crash response and care between 2018 and 2023

Based on our analysis of comparable indicators between the two reporting periods, an increase was observed in only one indicator. Availability of doctors with trauma surgery specialisation increased by 1% since 2018 while declines in reported availability were observed across the remaining indicators. Fewer countries reported a single national emergency number dropping from 21 (48%) in 2018 to 12 (27%) in 2023, and countries reporting national trauma registries have reduced from 34 (68%) to 19 (42%). 12 (27%) countries reported assessment of emergency systems in 2018 compared with 7 (16%) countries in 2023. Furthermore, the availability of specialised service providers in emergency medicine and emergency nurses is reported in fewer countries. (figure 3)

**Figure 3:**
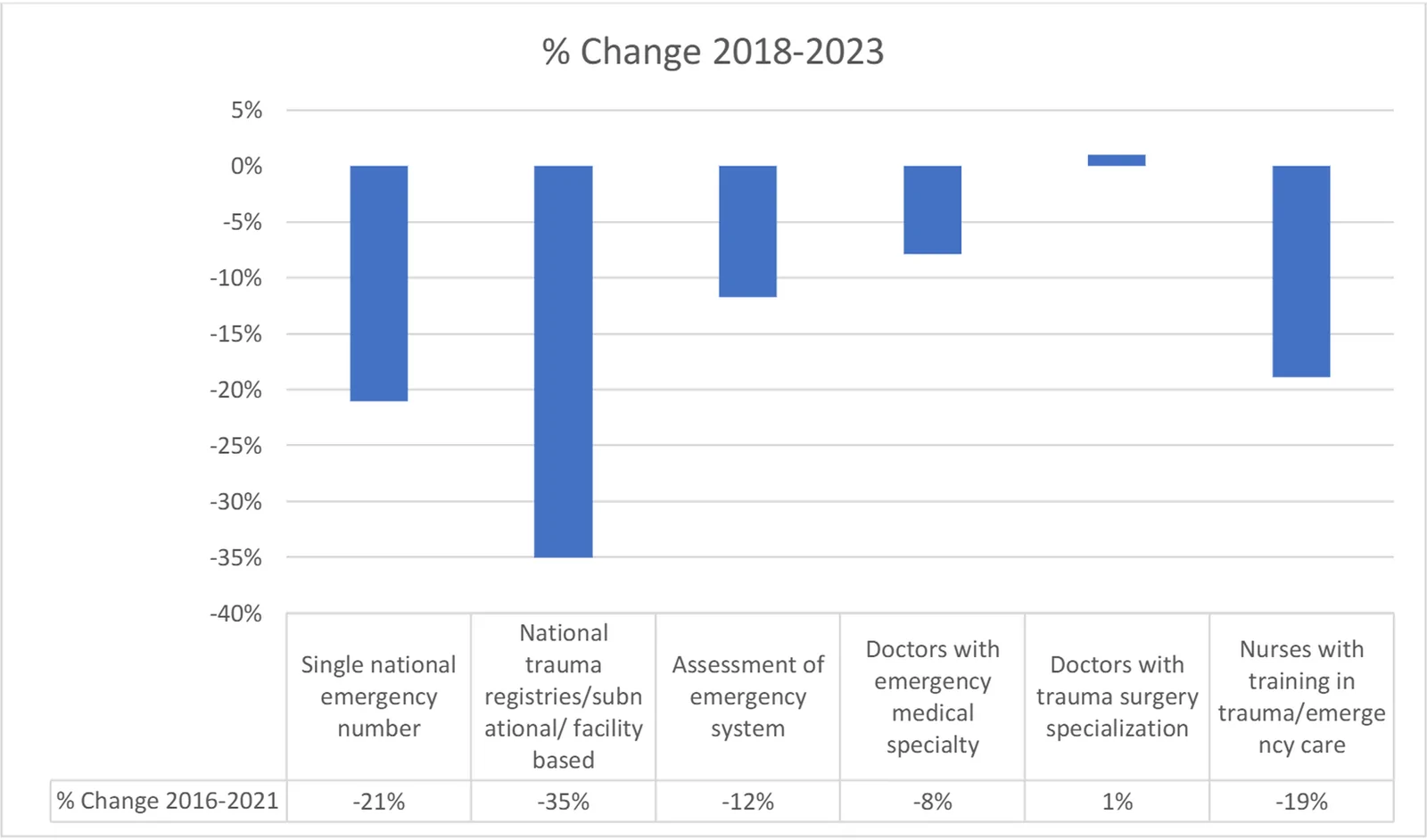
Change between 2018 and 2023 data on post-crash response indicators.

These observed changes should be interpreted with caution. Differences between 2018 and 2023 reporting periods may reflect variations in participating countries and differences in respondents and level of awareness, rather than true changes in system capacity. In addition, the 2023 data collection period coincided with the later stage of the COVID-19 pandemic, which may have disrupted emergency care services and affected both performance and reporting practices. As such, the reported declines in several indicators may not fully represent a true decline in post-crash response capacity across the region.

## Discussion

This study highlights a heavy burden of road traffic crashes in the African region. The regional reported fatality-to-crash ratio was nearly one death for every five reported crashes although substantial variation was observed across countries.

Post-crash response and care can be described as a series of critical steps that begin at the scene of the crash, to the trauma care facility and long-term care of the injured. At each step, providing timely and adequate care is essential to reduce mortality, morbidity and disability. This study provides the most up-to-date description of the situation of post-crash response in 45 Member States of the WHO African region and a brief comparison with the previous^16^ as it relates to post-crash response and care.

The findings suggest important gaps in post-crash response systems in the region, although large variations exist between countries. Key indicators show that relatively few countries report implementing elements aligned with recommended practices for post-crash response and care. Except for the presence of a national agency to coordinate prehospital and emergency care services in 80% of countries and national laws mandating third-party liability insurance for powered vehicles in 84% of countries, most indicators suggest limited reported availability of laws, services, or system components supporting prehospital and emergency care. Laws providing universal access to emergency care are implemented only in a handful of countries. Prehospital care, including access to an ambulance and access to qualified prehospital providers, appear limited in many countries. While most countries have an emergency number, multiple numbers that provide full coverage are more customary than the recommended practice of a single nationwide emergency number. Trauma care facilities, when available to meet demand, were more commonly reported in urban than rural settings. About half of the countries report having specialised trauma doctors. However, fewer report having specialised nurses. Free access to psychological support and rehabilitation services was reported in less than 10% of the countries. Compared to the Global Status Report on Road Safety 2018 findings, declines in the reported availability of several post-crash response indicators were observed.

These findings are in line with the scientific literature describing post-crash and trauma care as being scarce, ill-equipped in terms of human and material resources, and fragmented in low- and middle-income countries.^15^

The African region remains the WHO region with the highest fatality rate from road traffic injuries, and the number of fatalities has not reduced in the last 10 years, unlike in all other regions.^1,2^ Limitations in post-crash response systems may contribute to the high burden of mortality and disability associated with road traffic injuries in the region.^6,19^ Effective post-crash care is essential to reduce fatality from road traffic crashes. Evidence from several settings suggests that strengthening post-crash care systems may contribute to reductions in mortality and disability from road traffic injuries.^7,15^

Many studies highlight poor prehospital services, including timely access to ambulances and qualified prehospital providers or trained lay first responders.^19–21^ Some estimations indicate that less than 10% of the population has access to ambulance services in Sub-Saharan Africa.^7,17^ The shortage of skilled prehospital providers is also noted.^17^ In addition to a limited number of trained prehospital and trauma specialists, the quality of training is also one of the challenges crippling trauma care in the region.^22,23^ Nurses are recognised as the backbone of the health system, including emergency care, in many countries, but their specialisation in trauma care has been slow, and many countries lack formal education programmes.^24^

Prehospital care must be a critical component of the actions undertaken as several studies suggest that a substantial proportion of road traffic deaths in Africa occur during the prehospital phase.^23,25^ Indeed, timely emergency prehospital care to traffic accident victims at the crash scene and subsequent transportation to the health facility may reduce the probability of serious injury and deaths. While the strict ‘golden hour’ concept has been challenged in literature, substantial evidence continues to support the importance of timely trauma care with minimum delays following an injury.^26–30^ Death or severe injury risk is believed to increase after this period.^31^ Well-organised prehospital and emergency medical care significantly reduces death and disabilities.^15^ Lay first responders are usually the first to be present at the scene of a crash. The inadequate training of lay responders has been documented in Africa and challenges the principle of “do no harm”. Certainly, inadequate training of first responders may compromise the quality and safety of care provided at the scene of a crash and governments must ensure appropriate training is provided to lay first responders.^24^ In this context, the training of lay first responders has been evaluated in many countries and some studies show positive outcomes and cost-effectiveness.^32,33^

The continuum of care after acute trauma care is disrupted when victims have limited access to psychological support and rehabilitation. A study conducted in low- and middle-income countries shows a need for rehabilitation services as 74% of study participants reported difficulty in accomplishing daily tasks at 6 months of the crash.^4^ Another study conducted in Rwanda shows the high prevalence of disability among post-crash injury victims at 36%, affecting disproportionately socio-economically underprivileged groups. The researchers point to the lack of rehabilitation as a contributing factor to disability.^34^ Contrary to what has been witnessed in other countries of the region, research in Namibia shows good access to rehabilitation services for road crash victims. However, there remain challenges in access for people living in non-urban areas.^35^ This demonstrates the critical gap between needs and available services and indicates the necessity to invest in rehabilitation services and assistive technology to enable recovery and return to school or work.

Disparities between high-income and low- and middle-income countries, as well as disparities within countries, leading to inequities in the provision of emergency care in sub–Saharan Africa, are well documented.^22,23,36^ Alayande *et al*^23^ show geographical differences and urban vs rural disparities in availability and quality of services and point to South Africa as having the most mature system in sub-Saharan Africa. However, geographical and economic disparities in access still persist in South Africa as well.^23^

Road traffic deaths are a major health issue and pose societal and equity concerns. Road traffic injuries impose significant financial burdens on victims and their families, with morbidity and disability leading to catastrophic out-of-pocket expenses that perpetuate the cycle of poverty.^37–39^ Our study shows a high adoption by countries of national mandatory third-party liability insurance for powered vehicles, which aims to ensure financial protection for victims of road traffic crashes by providing immediate financial assistance to the injured following a crash. However, this assessment did not investigate the translation of the law into practice to effectively reduce the financial burden. On the other hand, our study reveals that few countries provide universal access to emergency care and free psychological and rehabilitation services, leaving many victims unprotected from these expenses. It is worth noting that road traffic deaths and injuries affect populations in the most productive years of life, further exacerbating the economic tragedy faced by families and communities.

Guidelines on effective interventions exist with clear recommendations on interventions and policies for effective post-crash response that save lives.^40^ From our study, we note that only a few countries are implementing the recommended actions. This trend has persisted despite the repeated global monitoring efforts through the WHO Global Status Reports on Road Safety. The uneven and incomplete progress suggests that, while global monitoring and benchmarking of post-crash care indicators has been strengthened, measurement and surveillance alone cannot drive implementation, particularly in low- and middle-income countries, without adequate governance, financing and implementation capacity.^1,41^ The specific challenges faced by African countries in implementing these recommendations are not fully established in the literature. Likewise, there is a need for more studies exploring the continuum of care, particularly the associations between road traffic crashes and long-term impairment and disability, and the broader implications of these outcomes on health and socio-economic equity.

While these findings point to important gaps in post-crash response systems, they should be interpreted with caution. The indicators used in this analysis reflect the reported availability of selected system components rather than their functionality, quality or effective coverage. As such, the presence or absence of policies or services does not necessarily translate into actual access to timely and appropriate care. Importantly, the findings highlight both policy gaps and implementation gaps. In some countries, the absence of legislation or formal systems indicates structural gaps, while in others, policies are in place but may not be effectively implemented, financed or accessible to the population. Distinguishing between these dimensions is critical for informing appropriate policy and system responses. The coexistence of relatively high levels of legislation mandating third-party liability insurance with limited access to emergency and rehabilitative services illustrates the complexity of post-crash response systems. This may reflect gaps in implementation, enforcement or financial protection mechanisms, rather than the absence of policy frameworks alone. In addition, post-crash outcomes such as mortality and disability are influenced by multiple factors beyond emergency care systems, including road infrastructure, injury severity, transport conditions and broader health system capacity. Variations observed across countries may therefore reflect differences in these contextual factors in addition to differences in post-crash response systems.

Strengthening multisectoral injury surveillance systems through improved integration of police, health facility, mortality and trauma registry data is also critical for improving the quality and completeness of post-crash data in the region. Previous studies in African settings have demonstrated substantial under-reporting in single-source systems and highlighted the value of multisource approaches for strengthening road traffic injury surveillance.^11,42^

Addressing these gaps will require prioritisation of feasible and context-specific interventions. Strengthening basic emergency care capacity, expanding training for lay and professional first responders and improving coordination across pre-hospital and facility-based services represent practical entry points in many resource-constrained settings.

### Strengths and limitations of the study

This study offers a regional overview of the key indicators related to post-crash response and care across 45 Member States of the African Region. The use of standardised WHO Global Status Report data and a multisectoral review and validation process strengthens the breadth and consistency of the information presented. By covering a wide geographical scope and a broad range of indicators, the study provides important insights into the reported availability of prehospital, emergency, rehabilitation and post-crash care system components across the region.

A key limitation of this study is the high proportion of missing data and ‘Do not know’ responses for several indicators. These responses may indicate limited availability of information at the national level, rather than the absence of services, and may reflect weaknesses in data systems or variations in respondent knowledge. This introduces uncertainty in the interpretation of some findings and limits the interpretation of some findings regarding the availability and coverage of post-crash response services.

The study also relies on country-reported data, which may reflect respondent knowledge rather than actual system capacity and may be subject to reporting bias. High levels of missing data and ‘do not know’ responses further limit interpretation. In addition, road traffic injury data in many countries rely heavily on police reporting systems, which are known to under-report injuries and deaths, particularly those occurring after hospital admission. These findings highlight the importance of strengthening multisectoral injury surveillance systems and improving integration of police, health facility and mortality data sources.

As a descriptive cross-sectional analysis, this study does not assess policy implementation or effectiveness and cannot establish causal relationships between system characteristics and outcomes. The descriptive design and the nature of the available country-level data did not permit assessment of causal relationships or adjustment for contextual factors such as economic status, urbanisation or broader health system capacity, which may influence post-crash outcomes and service availability. This is similar to most post-crash literature, with relatively few studies that have used designs or datasets capable of evaluating intervention effectiveness or establishing causal inference. Recent reviews of road safety and post-crash interventions in low-and-middle-income countries found limited studies with designs or data sufficient to permit causal inference or estimate intervention effects.^43^ This likely reflects the complex and content-dependent nature of post-crash systems, involving multiple sectors and levels of care, making standardisation across settings challenging.^40,44,45^ Furthermore, the cross-sectional and heterogenous structure of available post-crash datasets limits their suitability for longitudinal analyses and causal evaluation.^43^

Moreover, this study does not permit the identification of barriers and facilitators to the effective implementation of post-crash response services, a critical aspect that requires further contextual and implementation-focused research. Understanding these contextual factors is essential for designing and implementing interventions that are tailored to country and regional contexts within the African Region. Future studies should address these gaps to provide a more comprehensive understanding of the factors influencing the effectiveness of post-crash response services in the region.

Consequently, further analytical studies using more detailed and standardised datasets are needed to better understand the relationship between post-crash system characteristics and road traffic injury outcomes in the region.

## Conclusion

Building on previous reports, our analysis suggests persistent gaps in post-crash response systems across the WHO African Region. Strengthening emergency care system assessments, expanding training for prehospital and emergency care providers, improving trauma and injury surveillance systems and enhancing coordination between police and health data systems should be prioritised to improve post-crash response in the region.

With only a few years remaining to achieve the Sustainable Development Goals (SDGs) by 2030, strengthening post-crash response systems remains an important component of efforts to reduce road traffic mortality, disability and related inequities in the WHO African Region.

## Supplementary material

10.1136/bmjph-2025-004541 online supplemental file 1

10.1136/bmjph-2025-004541 online supplemental file 2

## Data Availability

Data are available in a public, open access repository. Data are available upon reasonable request.
