# Supplementary material for: Status of post-crash response systems and policy indicators in the WHO African region: a cross-sectional descriptive study
Source: BMJ Public Health. 2026 Oct 9;4(Suppl 1):e004541. doi: 10.1136/bmjph-2025-004541 (PMC13656106; doi:10.1136/bmjph-2025-004541)
Supplement: Supplementary file 1 [file bmjph-4-Suppl_1-s001.pdf]

Supplement 1

Global Status Report on Road Safety 2023 report		
Participating country, n=45	Income level	Subregion
Algeria	Lower middle income	West
Benin	Lower middle income	West
Botswana	Upper middle income	South
Burkina Faso	Low income	West
Burundi	Low income	East
Cabo Verde	Lower middle income	West
Cameroon	Lower middle income	Central
Central African Republic	Low income	Central
Chad	Low income	Central
Comoros	Lower middle income	East
Congo	Lower middle income	Central
Côte d'Ivoire	Lower middle income	West
Democratic Republic of the Congo	Low income	Central
Eritrea	Low income	East
Eswatini	Lower middle income	South
Ethiopia	Low income	East
Gabon	Upper middle income	Central
Gambia	Low income	West
Ghana	Lower middle income	West
Guinea	Low income	West
Guinea-Bissau	Low income	West
Kenya	Lower middle income	East
Lesotho	Lower middle income	South
Liberia	Low income	West
Madagascar	Low income	South
Malawi	Low income	South
Mali	Low income	West
Mauritania	Lower middle income	West

Mauritius	Upper middle income	South
Mozambique	Low income	South
Namibia	Upper middle income	South
Niger	Low income	West
Nigeria	Lower middle income	West
Rwanda	Low income	East
Sao Tome and Principe	Lower middle income	Central
Senegal	Lower middle income	West
Seychelles	High income	South
Sierra Leone	Low income	West
South Africa	Upper middle income	South
South Sudan	Low income	East
Togo	Low income	West
Uganda	Low income	East
United Republic of Tanzania	Lower middle income	East
Zambia	Low income	South
Zimbabwe	Lower middle income	South

Global Status Report on Road safety 2018<br>Participating countries n=44	
Angola	Lesotho
Benin	Liberia
Botswana	Madagascar
Burkina Faso	Malawi
Burundi	Mali
Cabo Verde	Mauritania
Cameroon	Mauritius
Central African Republic	Mozambique

Chad	Namibia
Comoros	Niger
Congo	Nigeria
Côte d'Ivoire	Rwanda
Democratic Republic of the Congo	Sao Tome and Principe
Equatorial Guinea	Senegal
Eritrea	Seychelles
Eswatini	South Africa
Ethiopia	South Sudan
Gabon	Togo
Gambia	Uganda
Ghana	United Republic of Tanzania
Guinea	Zimbabwe
Guinea-Bissau	
Kenya	
