# Supplementary material for: Status of post-crash response systems and policy indicators in the WHO African region: a cross-sectional descriptive study
Source: BMJ Public Health. 2026 Oct 9;4(Suppl 1):e004541. doi: 10.1136/bmjph-2025-004541 (PMC13656106; doi:10.1136/bmjph-2025-004541)
Supplement: Supplementary file 2 [file bmjph-4-Suppl_1-s002.docx]

| Country n=40 | Reported Number of Crashes | Reported Number of individuals Injured | Reported Number of Deaths | Reported fatality-to-crash ratio (%) |
| --- | --- | --- | --- | --- |
| Algeria | 23409 | 31649 | 3322 | 14% |
| Benin | 3666 | 5182 | 1124 | 31% |
| Botswana | 3008 | 4806 | 413 | 14% |
| Burkina Faso | 25000 | 15340 | 1272 | 5% |
| Burundi | 2289 | 3734 | 592 | 26% |
| Cabo Verde | 1066 | 1066 | 39 | 4% |
| Cameroon | 10977 | 2869 | 930 | 8% |
| Central African Republic | 3260 | 3839 | 1370 | 42% |
| Chad | 2704 | 3637 | 254 | 9% |
| Comoros | 286 | 371 | 32 | 11% |
| Congo | 1103 | 1927 | 223 | 20% |
| Côte d'Ivoire | 27531 | 33958 | 1614 | 6% |
| Democratic Republic of the Congo | 3364 | 4529 | 3364 | 100% |
| Eswatini | 2148 | 4001 | 229 | 11% |
| Ethiopia | 6417 | 10325 | 3971 | 62% |
| Gabon | 392 | 709 | 89 | 23% |
| Gambia | 712 | 903 | 200 | 28% |
| Ghana | 6701 | 14292 | 2890 | 43% |
| Guinea | 4430 | 3098 | 682 | 15% |
| Guinea-Bissau | 739 | 509 | 100 | 14% |
| Kenya | 10210 | 20625 | 4579 | 45% |
| Lesotho | 1277 | 1277 | 282 | 22% |
| Liberia | 1500 | 1719 | 232 | 15% |
| Madagascar | 1260 | 8686 | 300 | 24% |
| Malawi | 4305 | 6774 | 1444 | 34% |
| Mauritius | 2086 | 2802 | 108 | 5% |
| Mozambique | 942 | 1938 | 944 | 100% |
| Namibia | 3048 | 5340 | 540 | 18% |
| Niger | 7543 | 13 | 1152 | 15% |
| Nigeria | 9809 | 38073 | 6205 | 63% |
| Rwanda | 4159 | 5928 | 655 | 16% |
| Senegal | 19566 | 22549 | 877 | 4% |
| Seychelles | 2554 | 382 | 7 | 0% |
| Sierra Leone | 834 | 1331 | 336 | 40% |
| South Sudan | 5824 | 25181 | 350 | 6% |
| Togo | 7392 | 9376 | 680 | 9% |
| Uganda | 9070 | 13878 | 4159 | 46% |
| United Republic of Tanzania | 436 | 2452 | 1368 | 314% |
| Zambia | 10976 | 14489 | 2163 | 20% |
| Zimbabwe | 6858 | 10613 | 1902 | 28% |
| Total | 238851 | 340170 | 50993 | 21% |

**Supplementary Materials 2: Reported Road traffic crashes, injuries, deaths, and reported fatality-to-crash ratio**
